# Moderately delayed post-insult treatment with normobaric hyperoxia reduces excitotoxin-induced neuronal degeneration but increases ischemia-induced brain damage

**DOI:** 10.1186/2045-9912-1-2

**Published:** 2011-04-27

**Authors:** Benoit Haelewyn, Laurent Chazalviel, Olivier Nicole, Myriam Lecocq, Jean-Jacques Risso, Jacques H Abraini

**Affiliations:** 1 Centre Universitaire de Ressources Biologiques, Université de Caen, France; 2 ERT 1083, UMR 6232, Université de Caen Basse Normandie, CNRS, CEA, Centre CYCERON, B.P. 5229, Boulevard Henri Becquerel, 14074 Caen cedex, France; 3 Institut des Maladies Neurodégénératives, UMR 5293, Université de Bordeaux, CNRS; 4 Institut de Recherche Biomédicale des Armées, antenne Toulon, France; 5 Centre de Recherche Université Laval Robert-Giffard, Québec, Canada

## Abstract

**Background:**

The use and benefits of normobaric oxygen (NBO) in patients suffering acute ischemic stroke is still controversial.

**Results:**

Here we show for the first time to the best of our knowledge that NBO reduces both NMDA-induced calcium influxes *in vitro *and NMDA-induced neuronal degeneration *in vivo*, but increases oxygen and glucose deprivation-induced cell injury *in vitro *and ischemia-induced brain damage produced by middle cerebral artery occlusion *in vivo*.

**Conclusions:**

Taken together, these results indicate that NBO reduces excitotoxin-induced calcium influx and subsequent neuronal degeneration but favors ischemia-induced brain damage and neuronal death. These findings highlight the complexity of the mechanisms involved by the use of NBO in patients suffering acute ischemic stroke.

## Background

Acute ischemic stroke is one of the most common causes of death and long-term neurologic morbidity in the adult population. The primary cause of acute ischemic stroke is a significant disruption of cerebral blood flow through thromboembolism that leads to an oxygen and glucose deprivation for the cell and subsequent over-stimulation of the excitatory N-methyl-D-aspartate (NMDA) glutamatergic receptor whose post-synaptic activation is known as a critical event in neuronal death and brain damage induced by acute ischemic stroke [1,2]. The most common approved therapy of acute ischemic stroke today is thrombolysis, but unfortunately thrombolytic therapies are substantially limited to about 2% of stroke patients mainly due to some contraindications to treatment and most importantly to narrow therapeutic time window [3]. Alternatively, because tissue hypoxia plays a critical role in the primary and secondary events that lead to ischemia-induced neuronal death [4], tissue oxygenation with 100 vol% normobaric oxygen (NBO) is generally thought a logical stroke strategy that is often standard practice in acute ischemic stroke patients despite controversial results and the adverse potential of oxygen for exacerbating brain tissue damage particularly during reperfusion [5-7].

In the present report, to better understand the mechanisms of NBO on excitotoxic-ischemic insults, we studied the effects of NBO on *in vitro *and *in vivo *models of excitotoxic insult and ischemic insult in neuronal cell cultures, acute brain slices and rats. NBO treatment was given in conditions shown previously to allow neuroprotection by some inert gases such as xenon, nitrous oxide or helium [8-12]. We performed additional experiments in *in vitro *models of excitotoxic/ischemic insults. Our results indicate that NBO reduces excitotoxin-induced neuronal degeneration but favors ischemia-induced brain damage. These findings highlight the complexity of the mechanisms involved by the use of NBO in patients suffering acute ischemic stroke.

## Methods

### Animals

All animal-use procedures were in accordance with the framework of the French legislation on biomedical experimentation (agreement n° 14-27) and The European Communities Council Directive of 24 November 1986 (86/609/EEC). Before being used, rats and mice were housed at 21 ± 0.5°C, in Perspex home cages with free access to food and water. Light was maintained on a light:dark reverse cycle, with lights on from 8:00 pm to 8:00 am.

### NMDA-induced brain damage

On the day of surgery, rats were anesthetized with 1.5% halothane in oxygen alone, mounted on a stereotaxic apparatus, and allowed breathing spontaneously throughout the surgical intervention. A burr hole was drilled and a micropipette (~10 μm at the tip) was lowered into the right striatum (A: 0.6 mm, L: 3.0 mm, V: 5.8 mm, from the bregma) in order to allow injection of 50 nmol NMDA in 1 μL PBS (pH 7.4) over a 2-min period. After an additional 5-min period, the micropipette was removed, and the wounds sutured. During surgery, body temperature was kept at 37.5 ± 0.5°C. The rats waked up in their home cage after about 10 min, where they had free access to food and water. One hour after NMDA injection, the rats were treated with medical air (n = 7) or NBO (n = 12) as described below. Sham rats were given saline alone (n = 8).

### MCAO-induced cerebral ischemia

Rats were subjected to middle cerebral artery occlusion (MCAO)-induced transient cerebral ischemia for 60 min. Rats were anesthetized with 1.5% halothane in oxygen alone, and allowed breathing spontaneously throughout the surgical intervention. A midline neck incision was performed, and the right common carotid artery was exposed. After coagulation of the branches of the external carotid artery, a nylon thread (0.18 mm in diameter) including a self-made distal cylinder of 3 mm long and 0.38 mm diameter was inserted in the lumen of the external carotid artery, directed into the internal carotid artery up to the origin of the middle cerebral artery, and secured to the external carotid artery. During surgery, the animals were maintained normothermic at 37.5 ± 0.5°C using a feedback controlled thermostatic heating pad. After surgery, rats were awaked and allowed moving freely in their home cage with free access to food and water. One hour after MCAO, the nylon thread was removed under a short halothane anesthesia of no more than 10-min duration to restore blood flow. Then, after removal of the nylon thread, the rats were returned to their home cage for one additional hour before being treated with medical air (n = 10) or NBO (n = 9) as described below. Sham rats (n = 6) were subjected to the same protocol, except that the nylon thread was just introduced in the external carotid artery but not in the internal carotid artery; then after, sham rats were exposed to medical air for 3 h.

### Assessment of infarct size

Fifty hours after intrastriatal injection of NMDA or induction of MCAO, the rats were killed by decapitation under halothane anesthesia. The brain was rapidly removed, frozen in isopentane, and placed at -80°C. Coronal brain sections (20 μm) were cryostat-cut, mounted on slides, and stained with thionin as follows: slices were briefly immersed in water, stained with thionin, dehydrated with serial alcohol and cleared with xylene, and coverslipped with eukitt^® ^mounting. Brain sections colored with thionin were digitized on a PC computer. Then, volumes of NMDA-induced neuronal death and MCAO-induced brain infarction were analyzed with an image analyzer (ImageJ^® ^software, Scion corp., MD, USA) by two blinded observers. The observers' estimations were averaged. The lesioned areas were delineated by the pallor of staining in the necrotic tissue as compared to the surrounding healthy tissue; NMDA- and MCAO-induced brain damages were calculated by integration of the infarcted surfaces over the whole brain, corrected for tissue edema when needed (MCAO) by calculating and taking into account the ipsilateral/contralateral brain hemispheres ratio and expressed in mm^3 ^of infarction volume.

### Effects of NBO on NMDA-induced calcium influx in cultured neuronal cells

Mouse cortical cultures of neurons were prepared from 14- to 15-days old embryos as described previously [13]. Cerebral cortices were dissected, dissociated and cultured on 24-plates coated with 0.1 mg/ml poly-D-lysine (Sigma) and 0.02 mg/ml laminin (Invitrogen) in Dulbecco's modified Eagle medium (DMEM; Sigma) containing 5% fetal bovine serum (FBS), 5% horse serum and 2 mM glutamine (all from Sigma). Cultures were kept at 37°C in a humidified atmosphere containing 5% CO_2_. Neuronal cultures were used after 14 days in vitro. Cultures on glass bottom dish (N°0, coverglass 0.085-0.13 mm; Mattek Corporation; USA) were loaded for 45 min with 10 μM fura-2/acetoxymethyl ester (AM; F-1201, Invitrogen) and 0.2% pluronic acid and incubated for an additional 15 min at room temperature in HEPES-buffered saline solution containing (in mM) 116 NaCl; 5.4 KCl; 1.8 CaCl_2_; 0.8 MgSO_4_; 1.3 NaH_2_PO_4_; 12 HEPES; 5.5 glucose; and 10 μM glycine at pH 7.45. Experiments were made at room temperature with perfusion at 2 ml/min with a peristaltic pump on the stage of an inverted microscope equipped with a 100W Xenon lamp and oil immersion objective.

Fura-2 fluorescence emissions (>510 nm) evoked by excitation through narrow band-pass filters (340 ± 5 nm/380 ± 6.5 nm) housed in a computer-controlled filter wheel were recorded using an intensified CCD camera (Coolsnap EZ). Ratio images were acquired on a PC at a maximal time resolution of 2 s per successive measurement using Metafluor 6.3 software (Universal imaging Corporation, Chester, PA, USA). Measurements were obtained simultaneously from 30-40 neurons per randomly selected field (N = 3, n = 90-120 per condition). Changes in intracellular free Ca^2+ ^in cortical neurons were induced by rapid NMDA stimulations of 25 μM for 30 seconds each with the presence of medical air (controls) or NBO in the buffered saline solution.

### OGD-induced cell injury in brain slices

Rats were killed by decapitation. Then, coronal brain slices (400 μM thickness) including the striatum (anteriority: from + 1.2 to + 2 mm from Bregma) were cut using a tissue chopper (Mickie Laboratory Engineering Co., Gomshall, Surrey, UK), placed in a freshly prepared artificial cerebrospinal fluid (aCSF) containing (in mM): 120 NaCl, 2 KCl, 2 CaCl_2_, 26 NaHCO_3_, 1.19 MgSO_4_, 1.18 KH_2_PO_4_, 11 d-glucose, and 30 HEPES, and allowed to recover at room temperature for 45 min. Then, brain slices were placed at 36 ± 0.5°C into individual vials containing freshly prepared aCSF, saturated and continuously bubbled with 100 vol% oxygen (25 mL/min per vial). Following a 30 min period of stabilization, the incubation aCSF solution was renewed with oxygenated aCSF maintained at 36°C; the slices were then incubated for 1 h in order to allow recording basal levels of lactate dehydrogenase (LDH). While control slices were incubated for an additional 20-min period in the same conditions, those corresponding to the "ischemic" group were incubated in a glucose-free solution, saturated and continuously bubbled with 100 vol% nitrogen (OGD slices). After this 20-min period of OGD, to mimic reperfusion and treatment, the medium was replaced in all groups with freshly prepared aCSF solution, saturated and continuously bubbled with either medical air or 100 vol% oxygen. Then, the incubation aCSF solution was renewed every 1 h during the 3-h "reperfusion" period. OGD-induced neuronal injury was quantified by the amount of LDH released in the incubation solution samples every 1 h; values were summed to determine total levels of LDH released during the 3-h post-OGD reperfusion period. LDH activity was measured using a spectrophotometer at 340 nm in 50 μL of incubation medium by following the oxidation (decrease in absorbance) of 100 mL β-NADH (3 mg in 10 mL PBS) in 20 μL sodium pyruvate (6.25 mg in 10 mL PBS) using a microplate reader. The number of slices was n = 23-26 per condition.

### Gas pharmacology and treatment

Oxygen and air of medical grade were used. In vivo, freely-moving rats were treated for a 3-h period with either medical air (controls) or 100 vol% oxygen (NBO) at a flow rate of 6 L/min in a closed chamber (10 L vol.) fitted with a viewing window to allow observation. Such conditions allowed maintaining carbon dioxide less than 0.03 vol% and humidity about 60-70%. All the animals were treated with medical air or NBO according to a blinded procedure. This consisted in giving each group of animals a "secret" code that remained unknown to the experimenters in charge of assessing the rat's neurologic and histologic outcome until the end of the study. For in vitro experiments, neuronal cell cultures and acute brain slices were continuously perfused with a HEPES-buffered saline solution set at pH 7.4, previously saturated with either NBO or medical air.

### Statistical analysis

Data are given as mean ± sem. Date were analyzed using Mann-Whitney non-parametric unpaired U-test. The level of significance was set at *P *< 0.05.

## Results

### Effect of NBO on NMDA-induced neuronal death *in vivo*

**Figure 1:**
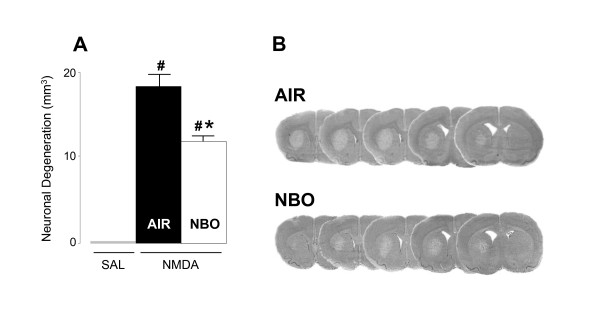
**Effect of NBO on NMDA-induced brain damage**. **(A) **Injection of NMDA led to significant brain damage as compared to sham rats injected with saline. Rats treated with NBO (n = 12) 1h after intrastriatal injection of NMDA showed a significant reduction of brain damage as compared to control animals treated with medical air (n = 7). **(B) **Typical brain slices obtained from rats injected with NMDA and treated with either medical air or NBO. Data are expressed as mean ± SEM (mm^3^). ^# ^*P *< 0.005 *vs *sham; * *P *< 0.01 *vs *NMDA control rats treated with medical air.

We investigated the effects of NBO in rats that were given an excitotoxic insult by intrastriatal infusion of 50 nmol NMDA. As illustrated in Figure 1, administration of NMDA in the striatum led to excitotoxic neuronal death of 18.3 ± 1.1 mm^3^. In line with previous findings that have shown redox modulation of the NMDA receptor with reduction producing a potentiation and oxidation an inhibition of the NMDA receptor response and glutamate-induced neuronal death [14,15], we found that NBO administered 1 h after NMDA injection reduced NMDA-induced neuronal death to 11.9 ± 0.7 mm^3 ^(*P *< 0.01), data corresponding to a decrease of neuronal death of about 35%.

### Effects of NBO on MCAO-induced brain damage

**Figure 2:**
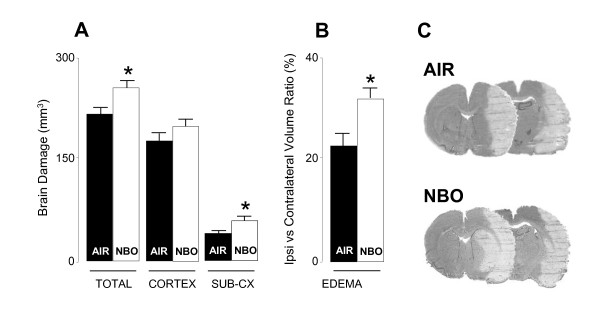
**Effects of NBO on MCAO-induced brain damage and behavioral motor deficits**. **(A) **NBO-treated rats (n = 9) had greater total and striatal damage, and showed a trend toward an increase in cortical brain damage, as compared with MCAO control rats treated with medical air (n = 10). Sham animals showed no brain damage (n = 6; data not shown). **(B) **NBO further increased brain edema and swelling as compared to MCAO control rats treated with medical air. **(C) **Typical examples of brain slices in MCAO rats treated with either medical air or NBO. Data are expressed as mean ± SEM (mm^3^). * *P *< 0.01 *vs *MCAO control rats treated with medical air.

Further, we studied the effects of NBO on MCAO-induced brain damage when given 1 h after MCAO induction. As shown in Figure 2, as compared to sham rats, MCAO control rats treated with medical air showed ischemia-induced brain damage (at both the cortical and subcortical level) and swelling of 218 ± 16 mm^3 ^and 23.9 ± 2.4%, respectively. NBO-treated rats had greater volume of total infarction of 255 ± 11 mm^3 ^(*P *< 0.01) and brain swelling of 32.2 ± 2.6% (*P *< 0.01), corresponding to a mean increase of respectively 17% and 34% as compared to MCAO control rats treated with medical air.

### Effect of NBO on NMDA-induced calcium influx and OGD-induced cell injury *in vitro*

**Figure 3:**
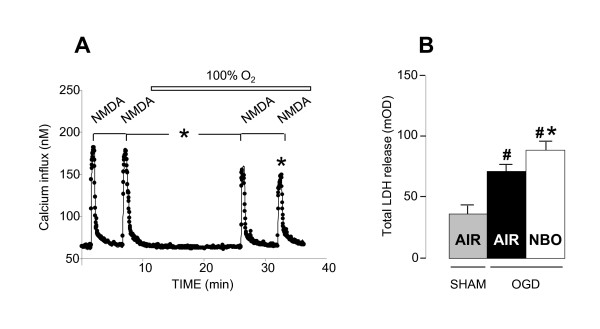
**Effects of NBO on NMDA-evoked Ca**^**2+ **^**influx in cortical neuronal cultures and OGD-induced LDH release in acute brain slices**. **(A) **Exposure to NMDA (25 μM) produces a rapid increase in intracellular free Ca^2+^, which recovered over the following minutes. NBO reduced NMDA-induced Ca^2+ ^influx by ~20%. For each experiment, N = 3, n = 90-120. **(B) **Exposure to OGD produces an increase in LDH release, as a marker of cell injury. NBO increases OGD-induced LDH release by ~25% as compared with OGD control slices treated with medical air. n = 23-26 per condition. Data are expressed as mean ± SEM. ^# ^*P *< 0.0005 *vs *sham; * *P <*0.001 *vs *NMDA or OGD.

Given the dichotomic effects of NBO on NMDA-induced neuronal degeneration and MCAO-induced brain damage in vivo, we performed additional experiments *in vitro *in neuronal cell cultures exposed to NMDA stimulations and brain slices exposed to experimental ischemia in the form of OGD. Exposure to 25 μM NMDA led to an important increase of 33% in intracellular free Ca^2+^, a process known as a key factor in excitotoxic neuronal death, which recovered over the following minutes. In line with our *in vivo *findings in rats injected with intrastriatal NMDA, we found that NBO slightly but significantly reduced NMDA-induced Ca^2+ ^influxes in neuronal cell cultures by about 17% (*P *< 0.001; Figure 3A). Alternatively, exposure to OGD led to an increase in LDH release as compared to sham slices. In line with our *in vivo *data in rats subjected to MCAO-induced ischemia, post-ischemic NBO treatment increased OGD-induced LDH release by 16 ± 0.3% (*P *< 0.001; Figure 3B).

## Discussion

In the present study, we investigated the effects of post-insult NBO treatment (given 1 h after reperfusion) on *in vivo *and *in vitro *models of excitotoxic-ischemic insult, namely brain damage induced by intrastriatal administration of NMDA, MCAO-induced cerebral ischemia, NMDA-induced calcium influxes in neuronal cell cultures, and cell injury produced by OGD in brain slices.

We found that post-insult NBO treatment reduced both NMDA-induced calcium influxes *in vitro *and NMDA-induced neuronal degeneration *in vivo*, but increased OGD-induced cell injury *in vitro *and MCAO-induced cortical brain damage *in vivo*. Taken together, these results indicate that post-insult NBO treatment reduces excitotoxin-induced calcium influx and subsequent neuronal degeneration but favors ischemia-induced brain damage and neuronal death. Previous investigations have demonstrated that NBO given during ischemia and/or immediately after ischemia may be a safe and effective stroke therapy [16-20] whereas in contrast NBO given during reperfusion may exacerbate brain tissue damage [6,21,22]. Given that NBO shows ability at reducing excitotoxin-induced neuronal death, which is well known to play a critical role in the development of ischemia-induced neuronal degeneration during the intra-ischemic period (before reperfusion), it is likely that the adverse effects of NBO at increasing ischemia-induced brain damage could occur through vascular-endothelial processes, which are known to be altered during and after reperfusion. Also, since both types of lesion are reduced by both intra- and post-ischemic inert gases that possess antagonistic properties at the NMDA receptor [9-12], the dichotomic effects of NBO on excitotoxin- and ischemia-induced neuronal death further indicate that NBO when given after reperfusion would favor some of the deleterious mechanisms that participate to neuronal death after a hypoxic/ischemic insult in a manner that overcomes its anti-excitotoxic properties. In that way, the adverse potential of oxygen for exacerbating tissue damage through the production of reactive oxygen species has been well documented particularly during reperfusion [5-7,21,22], but also discussed [16].

## Conclusions

In conclusion, it should be kept in mind that so far as today clinical trials with NBO have failed to show benefits in acute ischemic stroke patients [23,24]. By demonstrating for the first time to the best of our knowledge that NBO reduces excitotoxin-induced cell injury and as reported previously favors ischemia-induced cell injury when given after reperfusion, the present study suggests that NBO could be given to patients suffering acute ischemic stroke, and then stopped once reperfusion has occurred. Clinical trials with NBO could be designed in that way.

## Competing interests

The authors declare that they have no competing interests.

## Authors' contributions

All authors have read and approved the final manuscript. BH and LC performed the in vivo experiments. ON and ML. performed the in vitro experiments. ON, JJR, and JHA designed the study and wrote the manuscript.
